# Supplementary material for: Hydrodynamics of spike proteins dictate a transport-affinity competition for SARS-CoV-2 and other enveloped viruses
Source: Sci Rep. 2022 Jun 30;12:11080. doi: 10.1038/s41598-022-14884-6 (PMC9245886; doi:10.1038/s41598-022-14884-6)
Supplement: Supplementary file 1 — Supplementary Information. [file 41598_2022_14884_MOESM1_ESM.pdf]

Supporting information
Hydrodynamics of spikes proteins dictate a transport-affinity competition for SARS-CoV-2 and other enveloped viruses

Nicolas Moreno[1], nmoreno@bcamath.org
Daniela Moreno-Chaparro[1]
Florencio Balboa Usabiaga[1]
Marco Ellero[1,2,3], mellero@bcamath.org
[1]Basque Center for Applied Mathematics, BCAM.Alameda de Mazarredo 14 , Bilbao 48400, Spain
[2]IKERBASQUE, Basque Foundation for Science, Calle de Maria Dias de Haro 3, 48013, Bilbao,Spain
[3]Zienkiewicz Center for Computational Engineering (ZCCE), Swansea University, Bay Campus, Swansea,UK

List of Supplementary figures and tables

1. Rigid multiblob methodology.

2. Dimensionless diffusivity D_t and D_r

3. Dimensionless diffusivity D_t and D_r for virions with spherical or ellipsoidal envelope with spikes

4. Optimal resolution Determination

Supplementary Figure 1: blob radius ro overloaps for sphere and ellipsoids.
Supplementary Figure 2: Representation of a tetrahedron discretization.

Supplementary Table 1: Test resolution for a single sphere and ellipsoid.
Supplementary Table 2: Summary of the favorable resolution for a sphere and ellipsoid.
Supplementary Table 3: Resolution test of normalized ellipsoid envelope with N_s=26 tetra-rod spikes.
Supplementary Table 4: Different resolution of a discretized tetrahedron.
Supplementary Table 5: Summary of optimal resolutions of a tetrahedron.

5. Rotational and traslational diffusivity of SARS-CoV-2

Supplementary Table 6: Comparative translational and rotational diffusivities computed with D_t virion formula and real values of viscosity, temperature and energy for SARS-CoV-2.
Supplementary Table 7: Actual diffusion of SARS-CoV-2 virion per maximum and minimum spikes number 26 ± 15.

6. Random distribution

Supplementary Figure 3: Dimensionless rotational and translational diffusivities of random and uniform distribution of the spikes.

Supplementary Table 8: Fitting results of mean random of ten groups of data, and a single random set of results.

7. Size of the spikes

Supplementary Figure 4: Dimensionless rotational and translational diffusivity for different spikes length l_s/R. Supplementary Figure 5: Dimensionless rotational and translational diffusivity for different tetra-spikes radius R_{tetra}.

8. Virions with spikes

Supplementary Figure 6: Dimensionless rotational diffusivity for the virions.
Supplementary Figure 7: Dimensionless translational diffusivity for the virions.

Supplementary Table 9: Morphological parameters of different type of virions with spikes.
Supplementary Table 10: Spike parameters for the virions according to the type of the spikes and the measurement.
Supplementary Table 11: Comparative test with different parameters; All spikes distribution is random, the resolution is 14.5, the number and type of spikes are in Supplementary Table 9.
Supplementary Table 12: Comparative translational and rotational diffusivities computed with D_t virion formula and real values of viscosity, temperature and energy for all tested viruses.

9. Fitting of the excess diffusivity functions

Supplementary Figure 8: Schematic of the functional dependence of rotational diffusion coefficient with N_s.

Supplementary Table 13: Fitting constant numbers and Coefficient of determination.

10. Viruses binding affinity information.

Supplementary Table 14: Binding affinity and K_d for the viruses.

11. Mesh construction

Supplementary Figure 9: Blob representation of spheres using 12, 42, and 162 blobs in a uniform distribution. Supplementary Figure 10: Blob representation of tetrahedrons using 10, 34 and 130 blobs in a uniform distribution. Supplementary Figure 11: Blob representation of rods using 1, 3 and 7 blobs.

12. Time scales for virion binding

Figure 12: Time of transportation in the rotational movement τ_r in 5e-06 m of mucus.
Supplementary Figure 13: Time of transportation in the translational movement τ_t in 5e-06 m of mucus.
Supplementary Figure 14: Time of transportation ratio τ_t/τ_r.
Supplementary Figure 15: Dimensionless rotational diffusivity for different type of spikes.
Figure 16: Dimensionless translational diffusivity for different type of spikes.

1 Rigid multiblob methodology

In one of his celebrated papers, Einstein used the linear response theory to demonstrate that the translational diffusion coefficient of a colloid Ω is related to its translational mobility [1]. The same arguments can be easily extended to include the rotational diffusion [2]. In both cases the diffusion coefficients, D_t and D_r, are porportional to the the mobilities,

$$D_t = \frac{k_B T}{3}\mathrm{Tr}\left(\boldsymbol{M}_t\right), \quad D_r = \frac{k_B T}{3}\mathrm{Tr}\left(\boldsymbol{M}_r\right), \tag{1}$$

where $k_B T$ is the thermal energy and Tr denotes the trace operator. The mobility components yield the linear and angular velocities of a colloid ($\boldsymbol{u}$ and $\boldsymbol{\omega}$) in response to applied forces and torques ($\boldsymbol{f}$ and $\boldsymbol{\tau}$),

$$\begin{pmatrix} \boldsymbol{u} \\ \boldsymbol{\omega} \end{pmatrix} = \begin{pmatrix} \boldsymbol{M}_t & \boldsymbol{M}_c \\ \boldsymbol{M}_c^T & \boldsymbol{M}_r \end{pmatrix} \begin{pmatrix} \boldsymbol{f} \\ \boldsymbol{\tau} \end{pmatrix}. \tag{2}$$

For colloids larger than a few nanometers the mobility components can be calculated using the Stokes equations to a good approximation [3, 4]. In this limit, the fluid velocity and pressure, $\boldsymbol{v}$

and p, obey the Stokes equations with viscosity η

$$-\boldsymbol{\nabla} p + \eta \boldsymbol{\nabla}^2 \boldsymbol{v} = 0, \tag{3}$$

$$\boldsymbol{\nabla} \cdot \boldsymbol{v} = 0, \tag{4}$$

while for boundary conditions one can assume that the fluid velocity obeys the no-slip condition at the colloid's surface and that it decays to zero at infinity. We have assumed through this work that the virion, including its spikes, behaves like rigid body, thus the no-slip condition for a virion located at $\boldsymbol{q}$ is quite simple,

$$\boldsymbol{v}(\boldsymbol{r}) = \boldsymbol{u} + \boldsymbol{\omega} \times (\boldsymbol{r} - \boldsymbol{q}) \quad \text{for all } \boldsymbol{r} \in \partial\Omega. \tag{5}$$

These partial differential equations are closed by the balance of force and torque. The integral of the fluid traction, $-\boldsymbol{\lambda}$, over the surface of the virion balance the external forces and torques apply to the virion [5]

$$\int_{\partial\Omega} \boldsymbol{\lambda} \, \mathrm{d}S_r = \boldsymbol{f}, \tag{6}$$

$$\int_{\partial\Omega} (\boldsymbol{r} - \boldsymbol{q}) \times \boldsymbol{\lambda} \, \mathrm{d}S_r = \boldsymbol{\tau}. \tag{7}$$

To solve the Stokes problem and compute the mobilities we use the rigid multiblob method [6]. We discretize the surface of the virion with N markers or *blobs* of radius a and with position $\boldsymbol{r}_i$. The blobs are subject to constraint forces, $\boldsymbol{\lambda}_i$, that ensure the rigid motion of the whole virion. Evaluating the no-slip condition at the blobs, as in collocation methods, leads to a linear system of equations for the unknowns $\boldsymbol{u}$, $\boldsymbol{\omega}$ and $\boldsymbol{\lambda}_i$,

$$\boldsymbol{v}(\boldsymbol{r}_i) = \sum_{j=1}^{N} (\boldsymbol{M}_B)_{ij} \, \boldsymbol{\lambda}_j = \boldsymbol{u} + \boldsymbol{\omega} \times (\boldsymbol{r}_i - \boldsymbol{q}) \quad \text{for } i = 1, \dots, N, \tag{8}$$

$$\sum_{j=1}^{N} \boldsymbol{\lambda}_j = \boldsymbol{f}, \tag{9}$$

$$\sum_{j=1}^{N} (\boldsymbol{r}_j - \boldsymbol{q}) \times \boldsymbol{\lambda}_j = \boldsymbol{\tau}, \tag{10}$$

In the no-slip equation, Eq. (8), the blob mobility matrix $(\boldsymbol{M}_B)_{ij}$ couples the force acting on the blob j to the flow generated at the blob i. We use the regularized Rotne-Prager mobility

$$(\boldsymbol{M}_B)_{ij} = \left(\boldsymbol{I} + \frac{a^2}{6} \boldsymbol{\nabla}^2_{\boldsymbol{r}} \right) \left(\boldsymbol{I} + \frac{a^2}{6} \boldsymbol{\nabla}^2_{\boldsymbol{r}'} \right) \boldsymbol{G}(\boldsymbol{r}, \boldsymbol{r}') |^{\boldsymbol{r}=\boldsymbol{r}_i}_{\boldsymbol{r}'=\boldsymbol{r}_j}, \tag{11}$$

where $\boldsymbol{G}(\boldsymbol{r}, \boldsymbol{r}')$ is Green's function of the Stokes equation, i.e. the Oseen kernel. The Rotne-Prager mobility has a closed analytical expression [7, 8]. Given the forces and torques the linear system (8)-(10) can be solved to compute the particle velocities or, equivalently, the mobility matrix components from Eq. (2).

2 Dimensionless diffusivity $\bar{D}_t$ and $\bar{D}_r$

The theoretical translational diffusion of a sphere is

$$D_t = \frac{k_B T}{6\pi r\eta}, \tag{12}$$

and the rotational is

$$D_r = \frac{k_B T}{8\pi r^3\eta}. \tag{13}$$

Furthermore, the translational mobility is

$$\boldsymbol{M}_t = \frac{1}{6\pi r\eta}, \tag{14}$$

and the rotational mobility is

$$\boldsymbol{M}_r = \frac{1}{8\pi r^3\eta}. \tag{15}$$

The ratio between computed mobility $\boldsymbol{M}_t$ and theoretical mobility $\boldsymbol{M}_t^o$ is given by,

$$\bar{D}_t = \frac{\boldsymbol{M}_t}{\boldsymbol{M}_t^o} \tag{16}$$

for translational, and

$$\bar{D}_r = \frac{\boldsymbol{M}_r}{\boldsymbol{M}_r^o} \tag{17}$$

for rotational.

3 Dimensionless diffusivity $\bar{D}_t$ and $\bar{D}_r$ for virions with spherical or ellipsoidal envelope with spikes

3.1 Sphere with spikes

$\bar{D}_t$ and $\bar{D}_r$ of virions with spikes are calculated as the ratio between the mobility of the virions with spikes $\boldsymbol{M}_t$ for translational and $\boldsymbol{M}_r$ for rotational, divided by the single sphere $\boldsymbol{M}_t|_{\text{sphere}}$ and $\boldsymbol{M}_r|_{\text{sphere}}$ respectively,

$$\bar{D}_t = \frac{\boldsymbol{M}_t}{\boldsymbol{M}_t|_{\text{sphere}}}, \tag{18}$$

$$\bar{D}_r = \frac{\boldsymbol{M}_r}{\boldsymbol{M}_r|_{\text{sphere}}}. \tag{19}$$

The Eq.18 and Eq.19 are the computed dimensionless diffusivities for virions with spherical envelope.

3.2 Ellipsoid with spikes

Similar to the single ellipsoid and the sphere with spikes, in this case, $\bar{D}_t$ and $\bar{D}_r$ are the dimensionless ratio between computed mobility of the particle with spikes $\boldsymbol{M}_t$, $\boldsymbol{M}_r$ divided by the calculated mobility of the single ellipsoid $\boldsymbol{M}_t|_{\text{ellip}}$ and $\boldsymbol{M}_r|_{\text{ellip}}$

$$\bar{D}_t = \frac{\boldsymbol{M}_t}{\boldsymbol{M}_t|_{\text{ellip}}}, \tag{20}$$

and

$$\bar{D}_r = \frac{\boldsymbol{M}_r}{\boldsymbol{M}_r|_{\text{ellip}}}. \tag{21}$$

The Eq.20 and Eq.21 are the computed dimensionless diffusivities for virions with ellipsoid envelope.

4 Optimal resolution determination

We calculate an optimal resolution to simulate the virions computing the rotational and translational mobilities of a sphere, ellipsoid, and different spikes morphologies. The optimal resolution is given when the difference between simulations converges. The resolution is given for the radius of the sphere divided by the distance between particles (ro)

$$\text{Resolution} = \frac{\text{Radius}}{ro}. \tag{22}$$

We test six different resolutions of 0.9 (12 particles), 1.8 (42 particles), 3.6(162 particles), 7.2(642 particles), 14.5(2562 particles) and 29 (10242 particles).

The errors of $\bar{D}_t$ and $\bar{D}_r$ are

$$\text{Error}_{D_t} = \frac{\boldsymbol{M}_t^o - \boldsymbol{M}_t}{\boldsymbol{M}_t}$$

and

$$\text{Error}_{D_r} = \frac{\boldsymbol{M}_r^o - \boldsymbol{M}_r}{\boldsymbol{M}_r},$$

in Supplementary Table 1. we registered the error for all the resolutions, and choose the favorable resolution as the best trade off between accuracy and computational cost. The resolution of 29 provided the better approximation, with a rather high computational cost. Therefore, we selected as a reasonable resolution the corresponding 14.5, with errors on the order of 1% in $\bar{D}_t$, and 3% for $\bar{D}_r$, for an spherical envelope. We remark that acceptable results can be already obtained with resolutions of 7.2, having errors of 2% for $\bar{D}_t$, and 6% for $\bar{D}_r$.

4.1 Ellipsoid - Equivalent radius R_e

For ellipsoids, we calculated an equivalent radius R_e of a sphere with equivalent volume. The volume of an ellipsoid with principal axis length a, b, and c, is

$$V_{\text{ellip}} = \frac{4}{3}\pi abc. \tag{23}$$

The equivalent radius is then given by

$$R_e = \left(\frac{3}{4\pi}V_{\text{ellip}}\right)^{\frac{1}{3}}. \tag{24}$$

4.2 Optimal resolution test

To determine an optimal resolution, are tested six different resolutions for sphere and ellipsoid envelopes. To calculate the mobility is important to consider the blob radius for the spheres and ellipsoids (section 4.2.1).

Supplementary Table 1: Test resolution for a single sphere and ellipsoid

**Resolution**	**Number of particles**	**Envelope**	M_t	M_r	R_e	$\frac{M_t^o - M_t}{M_t}$	$\frac{M_r^o - M_r}{M_r}$	**Figure**
29	10242	Sphere	0.0527	0.0391	-	0.005	0.016	-
14.5	2562	Sphere	0.0524	0.0384	-	0.012	0.035	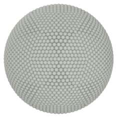
7.2	642	Sphere	0.0518	0.0372	-	0.024	0.065	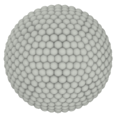
3.6	162	Sphere	0.0503	0.0304	-	0.052	0.234	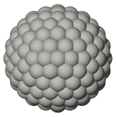
1.8	42	Sphere	0.0472	0.0297	-	0.110	0.254	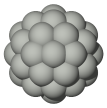
0.9	12	Sphere	0.0420	0.0213	-	0.208	0.465	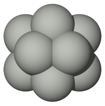
29	10242	Ellipsoid	0.0609	0.0595	0.8671	0.004	0.025	-
14.5	2562	Ellipsoid	0.0600	0.0570	0.8770	0.008	0.033	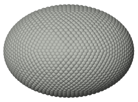
7.2	642	Ellipsoid	0.0585	0.0534	0.8967	0.012	0.033	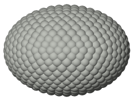
3.6	162	Ellipsoid	0.0550	0.0460	0.9360	0.030	0.051	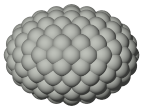
1.8	42	Ellipsoid	0.0476	0.0323	1.0134	0.091	0.156	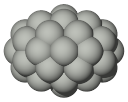
0.9	12	Ellipsoid	0.0392	0.0152	1.157	0.144	0.406	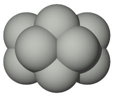

Supplementary Table 2: Summary of the favorable resolution for a sphere and ellipsoid

Test	Envelope	Resolution	R_x	R_y	R_z	R_t	R_r	$\boldsymbol{M}_t$	$\frac{\boldsymbol{M}_t^o - \boldsymbol{M}_t}{\boldsymbol{M}_t}$	$\boldsymbol{M}_r$	$\frac{\boldsymbol{M}_r^o - \boldsymbol{M}_r}{\boldsymbol{M}_r}$
**1**	Sphere	14.5	1	1	1	1.057	1.050	0.052	0.012	0.038	0.035
**2**	Ellipsoid	14.5	1	0.9	0.7	0.927	0.923	0.060	0.008	0.057	0.033

4.2.1 Blob radius

In the computational methodology RMB, we consider particles are located at a distance r_o, and set a blob radius of $r_b = ro/2$ for spheres and $r_b = 0,75ro$ for ellipsoids to ensure the accuracy of the method.

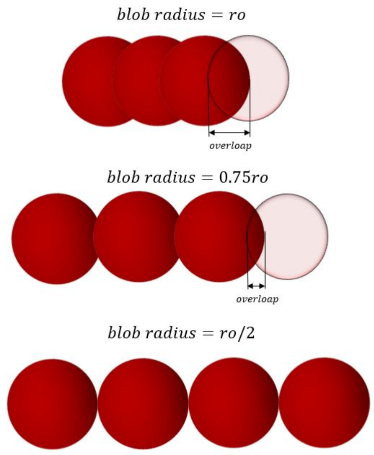

Supplementary Figure 1: blob radius ro have overloaps, to minimize the overloap is defined a blob radius of $ro/2$ for sphere, and $0.75ro$ for ellipsoids.

4.3 Resolution envelope and spikes for SARS-CoV-2

We test four different resolution using spikes and envelope with the SARS-CoV-2 configuration. The main is identify the resolution differences using the spikes and envelope together. In Supplementary Table 3, is illustrated the outcomes for the dimensionless diffusivity and the computed radius. Considering the resolution of 29 as the best approximation, an optimal resolution of 14.5 have a maximum error of 0.7%. This is in contrast with coarser resolutions of 3.6 with errors on the order of 20% for $\bar{D}_r$.

Supplementary Table 3: Resolution test of normalized ellipsoid envelope with N_s=26 tetra-rod spikes. The spikes length is l_s/R=0.5 and tetrahedron width is w_s/R=0.31. Where R is the largest axis of the ellipsoidal envelope.

**Resolution**	$\bar{D}_t$	$\bar{D}_r$	R_t	R_r	**Figure**
29	0.7682	0.4162	1.133	1.171	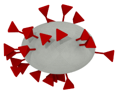
14.5	0.7638	0.4131	1.157	1.190	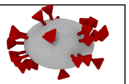
7.2	0.7478	0.3882	1.213	1.243	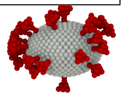
3.6	0.7075	0.3303	1.364	1.378	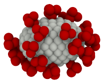

4.4 Tetrahedral spike resolution

For tetrahedral spikes we also conduct a separate calibration of the tetrahedron alone. We consider a tetrahedron of with $a = w_s$, inter-blob distance r_o, height is $H_{\text{tetra}} = \frac{\sqrt{6}}{3}a$, and circumscribed in a sphere of radius $R_{\text{tetra}} = w_s(3/8)^{1/2}$ See Supplementary Figure 2. Then, we characterize them using its equivalent radius (R_e), defined by the radius of a sphere with equivalent volume of the tetrahedron, $V_{tetra} = \frac{a^3}{6\sqrt{2}}$, leading to

$$R_e = \left(\frac{3}{4\pi}V_{\text{tetra}}\right)^{\frac{1}{3}}. \tag{25}$$

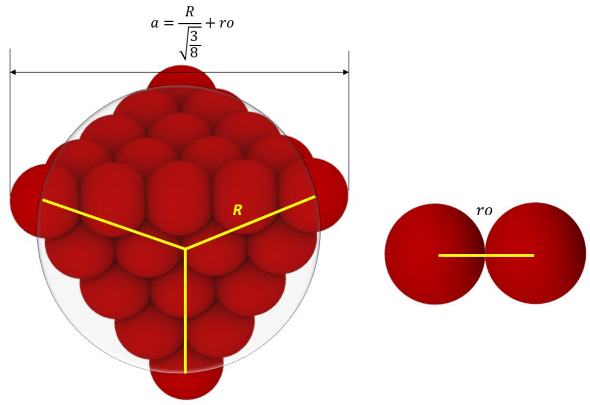

Supplementary Figure 2: Representation of a tetrahedron of width a, inter-blob distance r_o, and circumscribed radius $R_{\text{tetra}} = w_s(3/8)^{1/2}$.

The diffusivities for the single spikes are reduced as for the spheres and ellipsoids, with respect to a reference mobility $\boldsymbol{M}_t^o$, such that

$$\bar{D}_t^{tetra} = \frac{\boldsymbol{M}_t^{\text{tetra}}}{\boldsymbol{M}_t^o},$$

$$\bar{D}_r^{tetra} = \frac{\boldsymbol{M}_r^{\text{tetra}}}{\boldsymbol{M}_r^o}.$$

Since we do not count with an analytical expression for the mobility of a tetrahedron, we define the reference mobility, as the one of a sphere of radius $R_e|_{\text{solid}}$, where $R_e|_{\text{solid}}$ is the equivalent radius of a solid tetrahedron of width a. In Supplementary Table 4, we present the computed mobilities for different resolutions R_{tetra}/r_o, and the convergence criteria for each case. As a convergence criteria, we use the relative difference with respect to the highest resolution achieved, before the calculation become computationally prohibitive. Using a spike resolution of 2.5 we obtain an error of 3% for M_t and 8% for M_r, further improvement is achieved with resolutions of 4.9 with M_t and M_r errors of 1% and 4% respectively. The better resolutions reduce the error, but, requires a higher computational cost.

Supplementary Table 4: Different resolution of a discretized tetrahedron. The mobilities for the different resolutions are reduced using the mobility of a sphere of radius R_e, for a solid tetrahedron of edge a. As a convergence estimator, we measure variation on the reduced mobility with respect to the largest resolution simulated.

Resolution	Number of particles	$\boldsymbol{M}_t/\boldsymbol{M}_t^o$	$\boldsymbol{M}_r/\boldsymbol{M}_r^o$	R_e	a	$\frac{\boldsymbol{M}_{t\max}-\boldsymbol{M}_t}{\boldsymbol{M}_{t\max}}$	$\frac{\boldsymbol{M}_{r\max}-\boldsymbol{M}_r}{\boldsymbol{M}_{r\max}}$	Figure
Solid tetrahedron		–	–	0.4966	1.6330	0	0	
40	8194	0.8215	0.4750	0.5042	1.6580	0	0	
19.6	2050	0.8200	0.4724	0.5121	1.6840	0.0019	0.0055	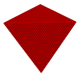
9.8	514	0.8159	0.4660	0.5276	1.7350	0.0069	0.0191	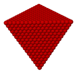
4.9	130	0.8074	0.4541	0.5587	1.837	0.0172	0.0440	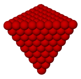
2.5	34	0.7918	0.4370	0.6207	2.0410	0.0362	0.0801	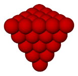
1.2	10	0.7651	0.4235	0.7448	2.4490	0.0688	0.1068	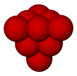

Supplementary Table 5: Summary of optimal resolutions of a tetrahedron. Low resolutions of 4.9 and 2.5 are the best options because of the computational cost of the simulations.

Resolution	Number of particles	Error$_{M_t}$	Error$_{M_r}$
4.9	130	1.7	4.4
2.5	34	3.6	8.0

5 Rotational and traslational diffusivity of SARS-CoV-2

For practical applications, virion diffusivities, $D_t|_{\text{virion}}$, can be easily retrieved using the known diffusivities of their respective plain envelope (sphere or ellipsoid), $D_i|_{\text{envelope}}$, and the reduced diffusivity $\bar{D}_t$, such that

$$D_t|_{\text{virion}} = \bar{D}_t D_t|_{\text{envelope}}, \quad (26)$$

$$D_t|_{\text{envelope}} = \frac{k_B T}{6\pi\eta R_{\text{env}}}. \quad (27)$$

For SARS-CoV-2, we compute the diffusivities for G-form and D-form, the results are in Supplementary Table 6.

Supplementary Table 6: Comparative translational and rotational diffusivities computed with D_t^{virion} formula and real values of viscosity, temperature and energy. The viscosity values are for water = 0.001 Pa · s, blood = 0.0035 Pa · s [9] and, nasal mucus = 1.6 Pa · s [10]. Temperature = 298.15 K, and kb = 1.38064852e-23 m^2kg/s^2K

	water		blood		nasal mucus	
Virus	D_t $[\mu m^2/s]$	D_r $[1/s]$	D_t $[\mu m^2/s]$	D_r $[1/s]$	D_t $[\mu m^2/s]$	D_r $[1/s]$
SARS-CoV-2 (G-form)	4.0451	965.4694	1.1557	275.8484	2.5281 e-3	0.6034
SARS-CoV-2 (D-form)	4.3345	1185.2682	1.2414	338.6481	2.7156 e-3	0.7408

Since the range in the number of spikes reported for SARS-CoV-2 is around 26 ± 15, for completness, we compute the diffusivities variation over that range as presented in Supplementary Table 7.

Supplementary Table 7: Actual diffusion of SARS-CoV-2 virion per maximum and minimum spikes number 26 ± 15

N_s	D_t $[\mu^2/s]$	D_r $[1/s]$
11	4.4842	1703.2178
26	4.0451	965.4694
41	4.0132	953.7624

6 Random distribution

The random distribution of spikes brings different mobility results. We compute ten replicas using the SARS-CoV-2 morphology with different number of spikes (12,15,18,21,24,26,30,33,36,39,42,50 and 100) and a resolution of 14.5, to estimate the error bars with the standard deviation. We calculate the fitting curve using the function in the Eq 28 of the mean of random replicas and just one of the replicas. The difference between the fitting functions illustrates an error of 2% in the number of spikes.

Supplementary Table 8: Fitting results of mean random of ten groups of data, and a single random set of results. The error is of 2% in the maximum number of spikes

**Mobility**	**TRANSLATIONAL**			**ROTATIONAL**		
	N_s^∞	b	$\bar{D}_t^\infty$	N_s^∞	b	$\bar{D}_r^\infty$
Mean random	141,6	10,7	0,670	105,2	6,1	0,304
Random	144,4	10,9	0,670	107,1	6,2	0,303
Error	1,9	1,8	0,000	1,7	2,5	0,330

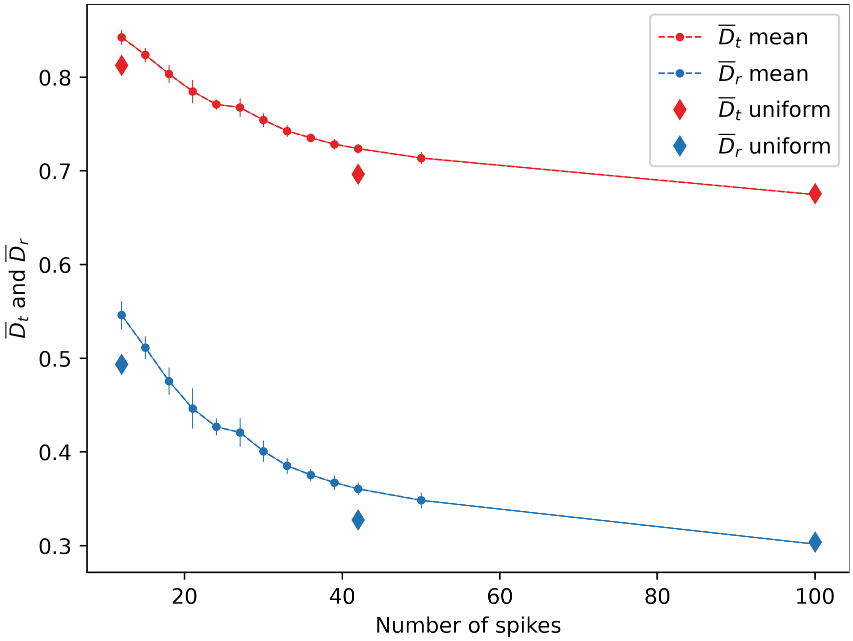

0

Supplementary Figure 3: Dimensionless rotational and translational diffusivities of random and uniform distribution of the spikes. For random distribution we obtain the mean and the error bars for 12,15,18,21,24,26,30,33,36,39,42,50 and 100 spikes. On the other hand, for uniform distribution we obtain diffusivities for 12, 42 and 162 spikes because the uniformity restrict the number of spikes. The values of 100 spikes is calculated with an interpolation between 42 and 162 values. However, it is evident that at large N_s, both random and uniform distribution converge due to the packing of the spikes in the envelope.

In Supplementary Figure 3 we present the dimensionless diffusivities of a SARS-CoV-2, using random and homogeneous distributions with different number of spikes. For the homogeneous distributions we required equidistant location of the spikes, thus we tested N_s equal to 12, 42, and 162. Models with 100 spikes cannot be constructed directly without further optimization on

spikes location, therefore for simplicity we calculated it as the interpolation between $N_s = 42$ and $N_s = 162$. For the random distribution are illustrated the mean of the diffusivities and the error bars. We can observe the error bars reduced as the number of spikes increases. Furthermore, the homogeneous distribution leads to diffusivities that are approximately 9.6% smaller than the random ones. However, as the number of spikes increases, the diffusivities of both homogeneous and random converged (difference of around 0.74%) due to the crowding of the spikes, leading to a close packing.

7 Size of the spikes

If the length and width of the spikes increase, $\bar{D}_t$ and $\bar{D}_r$ diminishes in a consistent fashion. Moreover, the rotational diffusivity exhibits a greater reduction than the translational one because of its dependency on the radius. Both, the length and the width are dimensionless, divided by the envelope radius. In Supplementary Figure 4 and 5, we present the variation on both $\bar{D}_t$ and $\bar{D}_r$ for virion models with rod and tetra spikes, respectively. For rod type the size l_s is varied, whereas for tetra type the radius R_{tetra} (see Supplementary Figure 2) is varied. We can observe that the dimensionality of the spikes affects the scaling of diffusion with the spike size. Comparing Supplementary Figure 4 and 5, rod shapes (that are dominantly one dimensional) showed a weaker variation on $\bar{D}_t$ and $\bar{D}_r$ as the size increases. In contrast, tetra-shape spikes displayed a strong reduction on diffusivity. Overall, we observed that the virions with bulkier and larger spikes (compared to the envelope size) had an intrinsic diffusional penalty. Therefore, the regulation on the number of spikes suggests a possible alternative to compensate for this reduction in mobility.

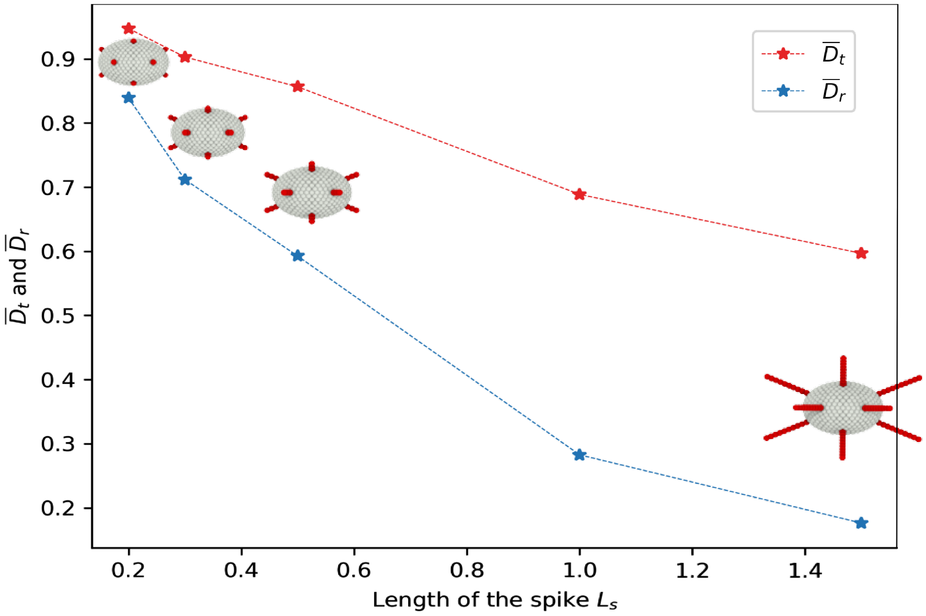

Supplementary Figure 4: Dimensionless rotational and translational diffusivity for different spikes length l_s/R. The simulation is with ellipsoid envelope, N_s=12, uniform distribution and rod spikes.

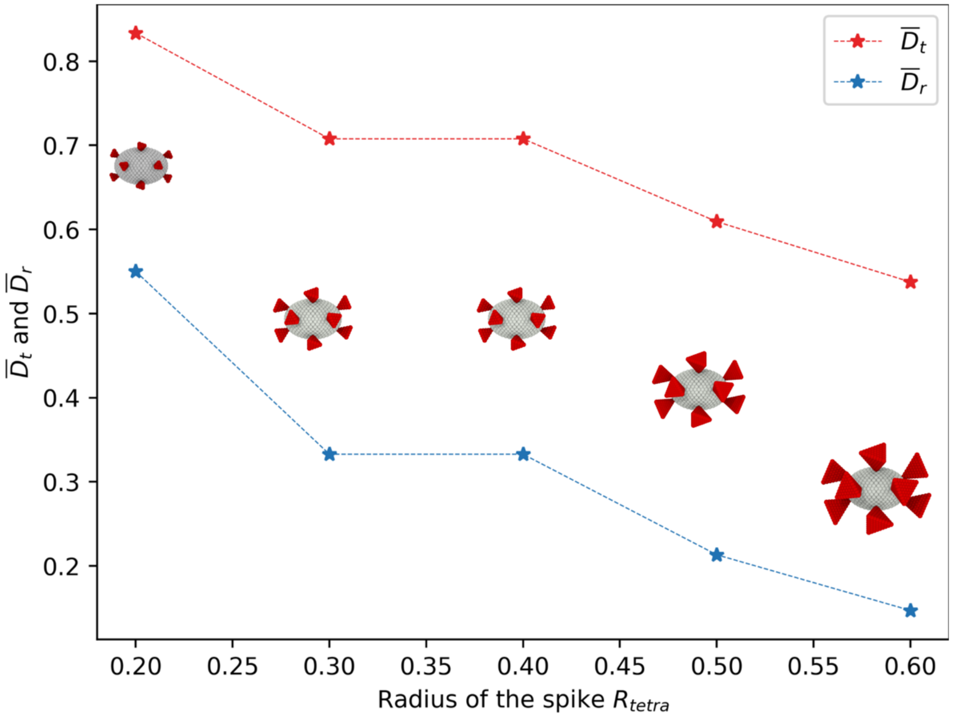

Supplementary Figure 5: Dimensionless rotational and translational diffusivity for different tetra-spikes radius R_{tetra}. The simulation is with ellipsoid envelope, N_s=12, uniform distribution and tetrahedron spikes.

8 Virions with spikes

Other viruses with similar morphologies as SARS-CoV-2 are tested and compared. The tested virions are SARS-CoV-2, HIV, MHV, SARS-CoV, Dengue, Lassa, Influenza and Herpes Simplex.

Supplementary Table 9: Morphological parameters of different type of virions with spikes

Virus	Radius envelope [nm]	Number of spikes	Length of spikes [nm]	Width of spikes [nm]	Type of spike	Reference
SARS-CoV2	48	26	25	15	Tetra rod	[11]
HIV	60	14	12	15	Tetra	[12]
Dengue	20	60	11	4	Tetra rod	[13]
Lassa	66	273	9	10	Sphere rod	[14]
Influenza	60	350	15	7	Rod	[15]
Herpes Simplex	93	659	20	5	Rod	[16]
SARS-CoV	50	65	16	18	Tetra	[17]
MHV	42.5	11	20	10	Sphere rod	[18]

Supplementary Table 10: Spike parameters for the virions according to the type of the spikes and the measurement. The dimensionless length and width are normalized with the radius of the virion, and are used to calculate the approximated total length of the spike $S\_s = (L\_s/R + W\_s/R + ro)$. S_s is used in the simulations.

Virions	Type of spike	Dimensionless Length L/R	Dimensionless Width W/R	S_s
SARS-CoV-2	Tetra rod	0,52	0,31	0,59
HIV	Tetra	0,20	0,25	0,34
Dengue	Tetra rod	0,55	0,20	0,57
Lassa	Sphere rod	0,14	0,15	0,34
Influenza	Rods	0,25	0,12	0,32
Herpes Simplex	Rods	0,22	0,05	0,27
SARS-CoV	Tetra	0,32	0,36	0,36
MHV	Sphere rod	0,47	0,24	0,51

Supplementary Table 11: Comparative test with different parameters. All spikes distribution is random, the resolution is 14.5, the number and type of spikes are in Supplementary Table 9.

**Test**	**Envelope**	R_x [nm]	R_y [nm]	R_z [nm]	R_t [nm]	R_r [nm]	$\bar{D}_t$	$\bar{D}_r$	Figure
**SARS-CoV-2**[11]	Ellips.	48	43	33	55,675	57,275	0,762	0,410	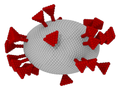
**HIV**[12]	Sphere	60	60	60	65,830	66,539	0,922	0,759	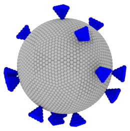
**Dengue**[13]	Sphere	20	20	20	27,039	27,532	0,749	0.397	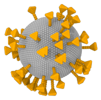
**Lassa** [14]	Sphere	66	66	66	78,227	78,442	0,854	0,617	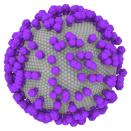
**Influenza** [15]	Sphere	60	60	60	70,253	70,472	0,864	0,6394	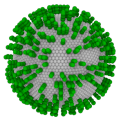
**Herpes S.** [16]	Sphere	93	93	93	105.000	105.129	0.896	0.717	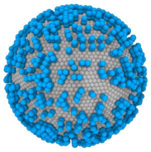
**SARS-CoV** [17]	Sphere	50	50	50	61.996	53.569	0.816	0.533	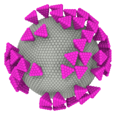
**MHV** [18]	Sphere	42.5	42.5	42.5	53.040	46.075	0.944	0.813	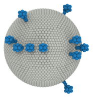

Diffusivities per number of spikes are illustrated in the Supplementary Figures 6 for rotational and Supplementary Figure 7 for translational.

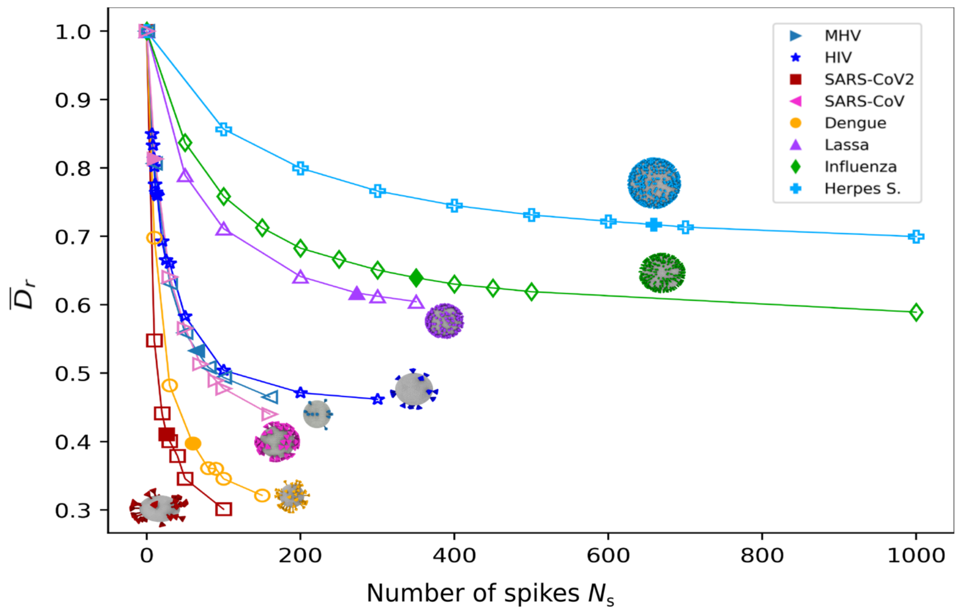

Supplementary Figure 6: Dimensionless rotational diffusivity for the virions. The coloured marker is the number of the spikes of the virion.

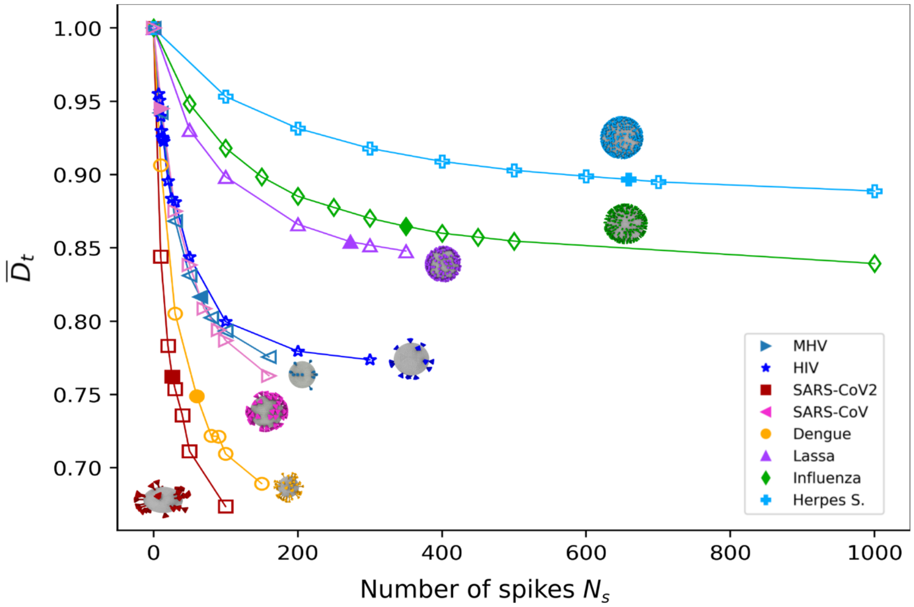

Supplementary Figure 7: Dimensionless translational diffusivity for the virions. The coloured marker is the number of the spikes of the virion

Supplementary Table 12: Comparative translational and rotational diffusivities computed with D_t^{virion} formula and real values of viscosity, temperature and energy. The viscosity values are for water = 0.001 Pa · s, blood = 0.0035 Pa · s [9] and, nasal mucus = 1.6 Pa · s [10]. Temperature = 298.15 K, and kb = 1.38064852e-23 $\text{m}^2\text{kg}/\text{s}^2\text{K}$

	water		blood		nasal mucus	
Virus	D_t $[\mu\text{m}^2/\text{s}]$	D_r $[1/\text{s}]$	D_t $[\mu\text{m}^2/\text{s}]$	D_r $[1/\text{s}]$	D_t $[\mu\text{m}^2/\text{s}]$	D_r $[1/\text{s}]$
SARS-CoV2	4.0451	965.4694	1.1557	275.8484	2.5281 e-3	0.6034
HIV	3.3594	576.2858	-	-	-	-
Dengue	8.1784	8127.9047	-	-	-	-
Lassa	2.8257	351.5049	-	-	-	-
Influenza	3.1483	484.5350	-	-	-	-
Herpes Simplex	2.1063	145.9985	-	-	-	-
SARS-CoV	3.852	843.828	-	-	-	-
MHV	4.532	1374.032	-	-	-	-

9 Fitting of the excess diffusivity functions

The results were plotted according the normalized diffusion $\bar{D}_t$ and $\bar{D}_r$. For example:

$$\bar{D}_i = \bar{D}_i^{\infty}(1 - \frac{(N_s^{\infty} - x)}{N_s^{\infty}}(1 - e^{\frac{-b}{x}})) + \frac{N_s^{\infty} - x}{N_s^{\infty}}(1 - e^{\frac{-b}{x}}), \tag{28}$$

where N_s^{∞} is the number of spikes at which the virion mobility saturates, b is a fitting parameter that depend on the characteristic size of the spike and, $\bar{D}_i^{\infty}$ is the diffusivity of the virus with me maximum number of spikes in the model simulation (Supplementary Figure 8).

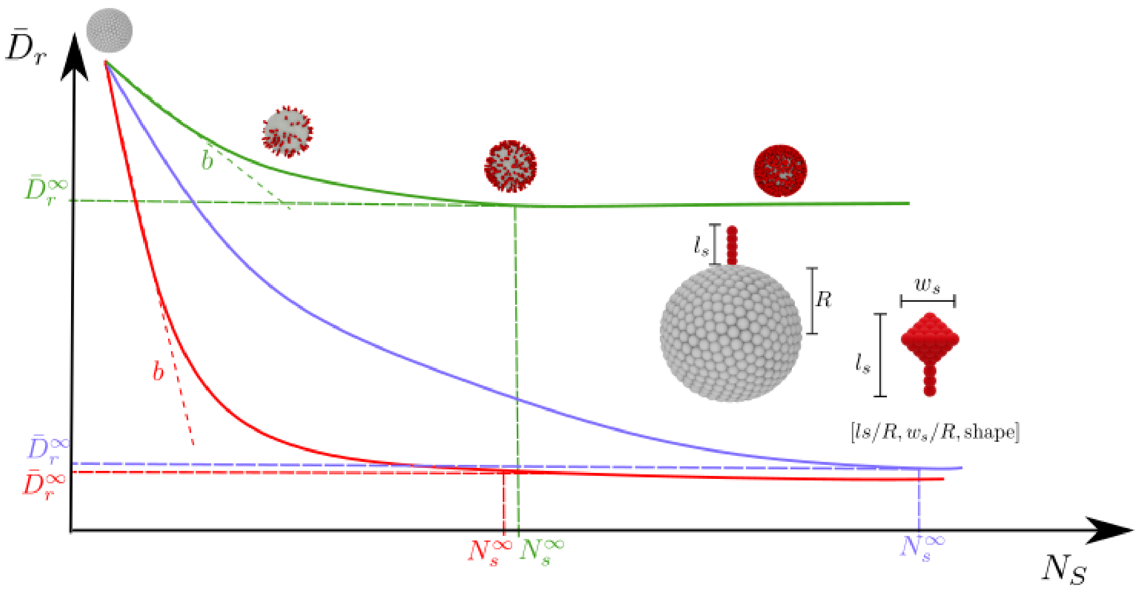

Supplementary Figure 8: Schematic of the functional dependence of rotational diffusion coefficient with N_s. The shape-dependent parameters N_s^{∞} and b characterize the behavior of the diffusional decay. N_s^{∞} indicates the saturation in mobility when the diffusion is no longer affected by N_s, whereas b sets the strength on the decay. Lower values of b correlates with bulkier spikes that induce a fast reduction in the mobility. Moreover the magnitude of b can be used as a differentiating parameter for virion classification.

To determine the accuracy of the proposed fitting equation we computed the coefficient of determination R_d^2. The mean $\bar{y}$ is

$$\bar{y} = \frac{1}{n}\sum_{i=1}^{n} y_i, \tag{29}$$

and the coefficient is

$$R_d^2 = 1 - \frac{\sum(y_i - f_i)^2}{\sum(y_i - \bar{y})^2}. \tag{30}$$

According the Eq.30 the calculated data f_i is the fitting function (Eq. 28), the observed data y_i are the dimensionless values of diffusion ($\bar{D}_i$), and the mean of the observed data $\bar{y}$ (Eq. 29) is for the dimensionless diffusion. The results summarized in Supplementary Table 13 show values of $R_d = 0.99$ between dimensionless diffusion and the fitting function introduced.

Supplementary Table 13: Fitting constant numbers Eq 28 N_s^∞, b, $\bar{D}_i^\infty$, and R^2 Coefficient of determination

		**TRANSLATIONAL**				**ROTATIONAL**			
Virus	N_s	$N_s^\infty \pm 2$	b ± 2	$\bar{D}_t^\infty$	R_d^2	$N_s^\infty \pm 2$	b ± 3	$\bar{D}_r^\infty$	R_d^2
SARS-CoV2 (G-form)	26 [11]	204.6	8.9	0.673	0.99	206.5	4.9	0.301	0.99
SARS-CoV2(D-form)	26 [11]	139.9	17.2	0.704	0.99	148.3	10.1	0.335	0.99
HIV	14 [12]	110.4	15.9	0.799	0.99	144.5	10.8	0.499	0.99
Dengue	60 [13]	229.3	14.7	0.689	0.99	196.9	9.1	0.321	0.99
Lassa	273 [14]	374.4	53.2	0.847	0.99	372.7	40.9	0.604	0.99
Influenza	350 [15]	579.3	70.3	0.854	0.99	580.9	54.1	0.619	0.99
Herpes Simplex	659 [16]	753.4	115.6	0.895	0.99	843.8	93.8	0.710	0.99
SARS-CoV	65 [17]	169.0	17.9	0.770	0.99	211.1	11.8	0.469	0.99
MHV	11 [18]	157.7	23.6	0.769	0.99	171.1	14.8	0.449	0.99

10 Viruses binding affinity information.

10.1 Spikes concentration

We approximate the molar concentration of spikes as $[S]$ =moles $\mathbf{S}/l$, to compute the saturation function $[S]/([S] + K_D)$ using the number of spikes N_s, the length of the spike l_s and the radius of the envelope R. The reaction volume is given by $4/3\pi((R + l_S)^3 - R^3)$, and the number of moles N_s/NA. In Supplementary Table 14, we summarize the magnitude of the binding affinities between spikes proteins and cell receptors for the different viruses modelled. Since the smaller the value of K_D, the smaller the amount of spikes needed to achieve an effective binding. Viruses with lower K_D will reach saturation earlier.

Supplementary Table 14: Binding affinity and K_d for the viruses

**Virus**	**Biding affinity**	K_d [M]	**Reference**
SARS-CoV-2(G-form)	ACE-2	0.3-15e-9	[19, 20]
SARS-CoV-2(D-Form)	ACE-2	0.6-11e-9	[19, 20]
HIV	CD4 with gp120	1.02e-9	[21]
Dengue	CLEC5A	172e-6	[22]
Lassa	GPC42-50,GPC60-68, and GPC441-449,	98e-9	[23]
Influenza	Ha-SA	10e-5	[24]
Herpes Simplex	gD and nectin-1	54e-9	[25]
SARS-Cov	ACE2	150e-9 to 300e-9	[26]
MHV	NTD	21.4 e-9	[27]

11 Mesh construction

11.1 Sphere

The construction of a sphere of radius R and distance between blobs r_b starts with the coarser representation of an icosphere with 12 vertex located at R and distance r_o between them, as shown in Supplementary Figure 9. One step refinement of this structure is conducted by taking the middle points of the segments connecting two adjacent vertex, and projecting those points radially, such that their new position satisfies $R^2 = x^2 + y^2 + z^2$. This refining procedure can be repeated until the distance between vertex is smaller or equal to the target inter-particle distance ($r_o \leq r_b$).

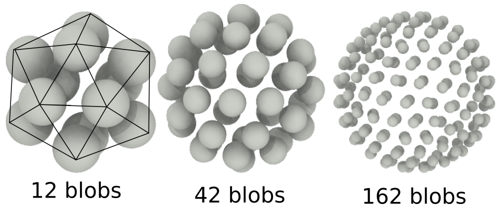

Supplementary Figure 9: Blob representation of spheres using 12, 42, and 162 blobs in a uniform distribution

11.2 Tetrahedron

The tetrahedron construction starts with the primary coarse surface with only four vertex. Following the same methodology that for the spheres, the structure is refined by splitting in half the edges

between two vertex and adding a new point in that position. This addition must be applied to all the edges of the primary surface. This procedure is repeated until the distance between adjacent points is smaller or equal that the target resolution. Tetrahedron with different three different resolutions are presented in SI.10

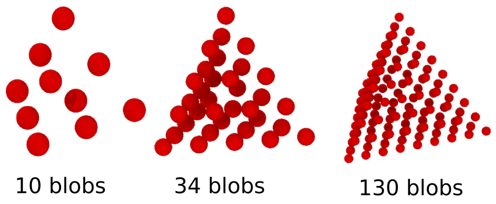

Supplementary Figure 10: Blob representation of tetrahedrons using 10, 34 and 130 blobs in a uniform distribution.

11.3 Rods

The rods construction consist in linking beads one by one, equidistant and all the centroids in the same angle.

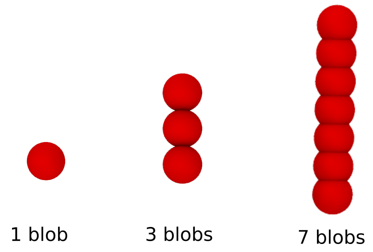

Supplementary Figure 11: Blob representation of rods using 1, 3 and 7 blobs

12 Time scales for virion binding

We compute the time of transportation of the virions in 5e-06 m of mucus, according the rotational and translational diffusivities. The results illustrated in Supplementary Figure 12 for rotational, and Supplementary Figure 13 for translational, show a dependency of the radius of the virion. For example, the biggest virion is the Herpes simplex, and the times (τ_t) and (τ_r) are the largest in contrast to the other virions transportation time.

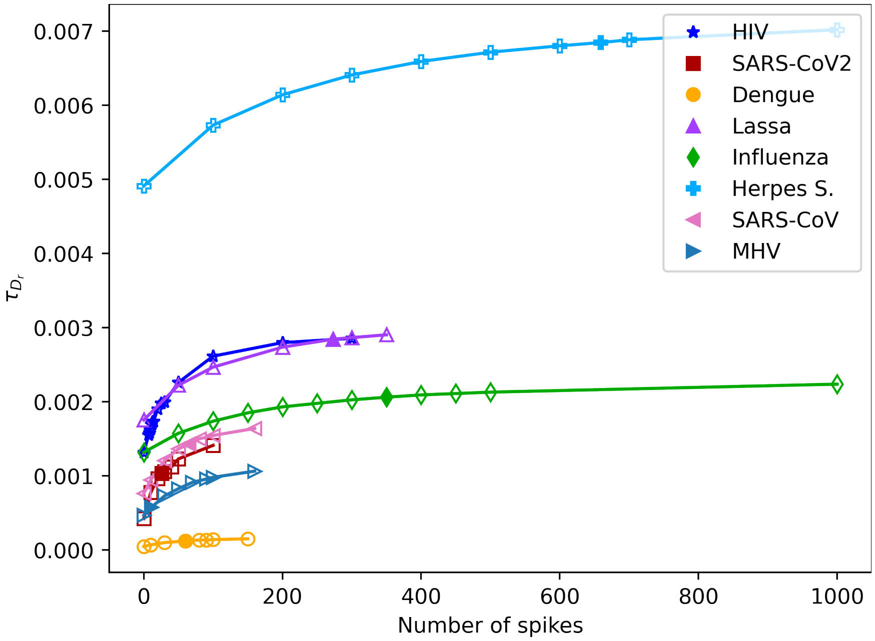

Supplementary Figure 12: Time of transportation in the rotational movement τ_r in 5e-06 m of mucus

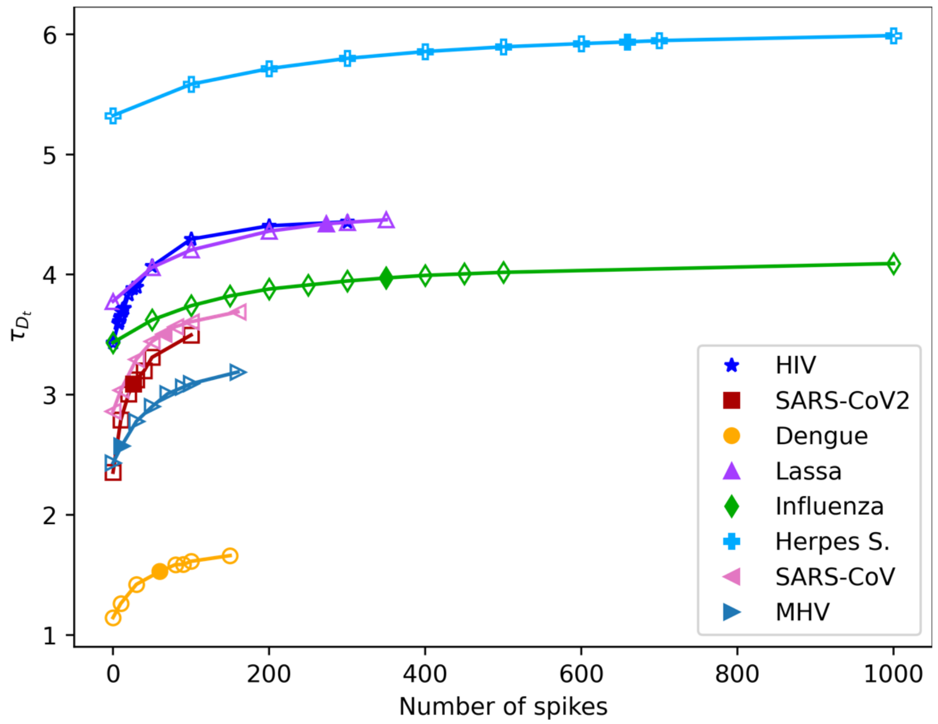

Supplementary Figure 13: Time of transportation in the translational movement τ_t in 5e-06 m of mucus

In Supplementary Figure 14 we have the ratio between the transportations time τ_t and τ_r, the results show a scale of 1000 seconds for the majority of the virions, but for the herpes simplex the scale is in 100 seconds and for dengue is for 10000 seconds. These differences are given by the radius of the virion.

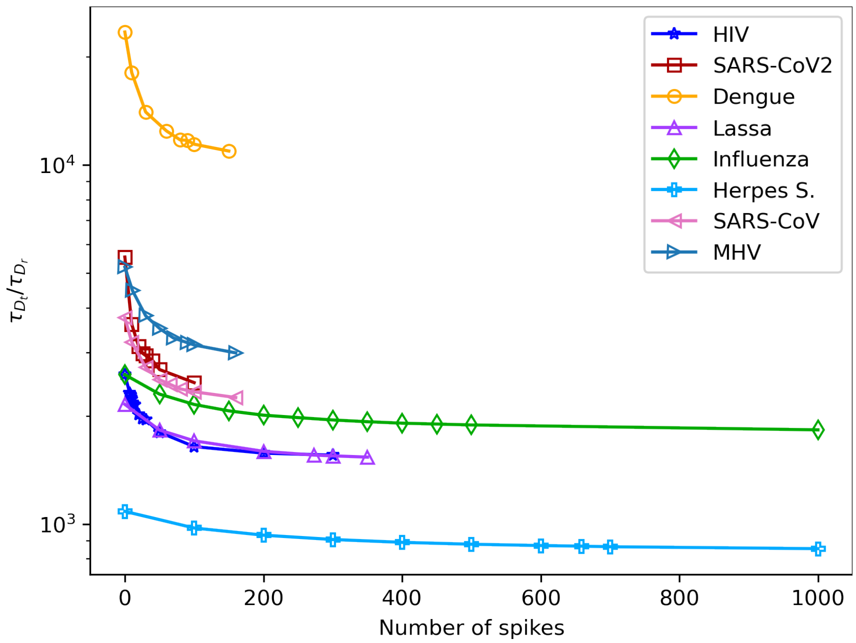

Supplementary Figure 14: Time of transportation ratio τ_t/τ_r

13 Type of spikes

Five types of spike morphologies are constructed using the RMB methodology: rod, tetra, sphere, rod-tetra, and rod-sphere. Rod, tetra, and sphere are characterize by a single lenght, whereas rod-tetra and rod-sphere require two. parameters

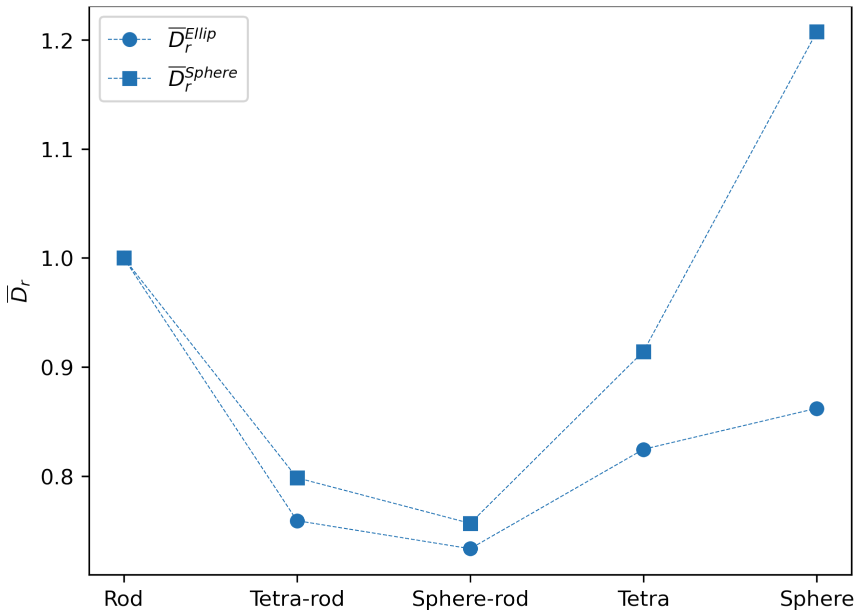

Supplementary Figure 15: Dimensionless rotational diffusivity for different type of spikes. Diffusivities are calculated using the ratio between the type of spike and the rod spike. Except for the sphere spike in a sphere envelope, all diffusivities are lower than the rod spikes.

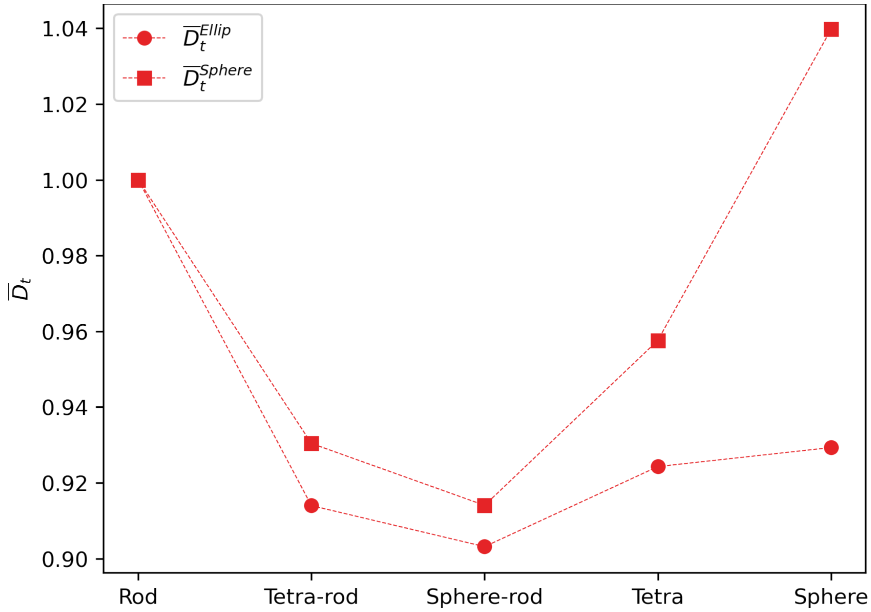

Supplementary Figure 16: Dimensionless translational diffusivity for different type of spikes. Diffusivities are calculated using the ratio between the type of spike and the rod spike. Except for the sphere spike in a sphere envelope, all diffusivities are lower than the rod spikes.

Supplementary Table 15: Parameters for simulation for different types of virus.

**Name**	**Symbol**
Biding constant affinity	K_D
Translational mobility for ellipsoid	$M_t\|_{\text{ellip}}$
Rotational mobility for ellipsoid	$M_r\|_{\text{ellip}}$
Translational mobility for tetra spike	$M_t\|_{\text{tetra}}$
Rotational mobility for tetra spike	$M_r\|_{\text{tetra}}$
Cell receptor	ϵ_{bind}
Coefficient of determination	R^2
Damkohler number	D_a
Dimensioneless rotational diffusivity	$\bar{D}_r$
Dimensioneless translational diffusivity	$\bar{D}_t$
Distance between points of the structure	ro
Energy values in molecular-scale systems	k_BT
Envelope	$\boldsymbol{E}$
Envelope translational diffusivity	$D_t\|_{\text{env}}$
Envelope rotational diffusivity	$D_r\|_{\text{env}}$
Equivalent radius	R_e
Geometrical constraint avidity	$\bar{\Gamma}$
Height of tetra spikes	H_{tetra}
Infectivity parameter	I
Lenght of the spike	l_s/R
Maximum number of spikes	N_s^{∞}
Number of spikes	N_s
Radius	R
Radius of the spike. Pamplomer radius	p_s/R
Reaction time	t_I
Reaction time biding	t_b
Reaction time diffusional	t_d
Rotational Diffusivity	D_r
Rotational virion diffusivity	D_r^{virion}
Spike proteins	$\boldsymbol{S}$
Temperature	T
Temperature of the blood	T_b
Theoretical rotational diffusivity	D_r^o
Theoretical translational diffusivity	D_t^o
Theoretical radius of the envelope	R_t^o
Time of rotational	T_b
Temperature of the blood	T_b

Traslational Diffusivity	D_t
Traslational virion diffusivity	D_t^{virion}
Viscosity	η
Viscosity of the blood	η_b
Volume of ellipsoid	V_{ellip}

References

[1] Albert Einstein. "On the movement of small particles suspended in stationary liquids required by the molecular-kinetic theory of heat". In: *Annalen der Physik* 17 (1905), p. 549.

[2] Sangtae Kim and Seppo J. Karrila. *Microhydrodynamics: principles and selected applications*. Ed. by Sangtae KimSeppo J. Karrila. Butterworth-Heinemann, 1991. ISBN: 978-0-7506-9173-4. DOI: http://dx.doi.org/10.1016/B978-0-7506-9173-4.50007-4. URL: http://www.sciencedirect.com/science/article/pii/B9780750691734500074.

[3] J. R. Schmidt and J. L. Skinner. "Brownian Motion of a Rough Sphere and the Stokes-Einstein Law". In: *The Journal of Chemical Physics B* 108 (2004), p. 6767. DOI: 10.1021/jp037185r.

[4] Florencio Balboa Usabiaga et al. "The Stokes-Einstein relation at moderate Schmidt number". In: *The Journal of Chemical Physics* 139 (2013), p. 214113. DOI: http://dx.doi.org/10.1063/1.4834696.

[5] C. Pozrikidis. *Boundary Integral and Singularity Methods for Linearized Viscous Flow*. Cambridge Texts in Applied Mathematics. Cambridge University Press, 1992. DOI: 10.1017/CBO9780511624124.

[6] F. Balboa Usabiaga et al. "Hydrodynamics of suspensions of passive and active rigid particles: a rigid multiblob approach". In: *Communications in Applied Mathematics and Computational Science* 11.2 (2016), pp. 217–296. DOI: 10.2140/camcos.2016.11.217.

[7] Jens Rotne and Stephen Prager. "Variational Treatment of Hydrodynamic Interaction in Polymers". In: *Journal of Chemical Physics* 50 (1969), p. 4831. DOI: http://dx.doi.org/10.1063/1.1670977.

[8] Eligiusz Wajnryb et al. "Generalization of the Rotne–Prager–Yamakawa mobility and shear disturbance tensors". In: *Journal of Fluid Mechanics* 731 (Sept. 2013), R3. DOI: 10.1017/jfm.2013.402. URL: http://journals.cambridge.org/article_S0022112013004023.

[9] Yongfei Jiang, Jun Zhang, and Wanhua Zhao. "Effects of the inlet conditions and blood models on accurate prediction of hemodynamics in the stented coronary arteries". In: *AIP Advances* 5 (2015), p. 57109. DOI: 10.1063/1.4919937. URL: http://dx.doi.org/10.1063/1.4919937].

[10] Jasmim Leal, Hugh D.C. Smyth, and Debadyuti Ghosh. "Physicochemical properties of mucus and their impact on transmucosal drug delivery". In: *International Journal of Pharmaceutics* 532.1 (Oct. 2017), pp. 555–572. ISSN: 0378-5173. DOI: 10.1016/J.IJPHARM.2017.09.018.

[11] Hangping Yao et al. "Molecular Architecture of the SARS-CoV-2 Virus". In: *Cell* 183.3 (2020), pp. 730–738. ISSN: 10974172. DOI: 10.1016/j.cell.2020.09.018. URL: https://doi.org/10.1016/j.cell.2020.09.018.

[12] Jun Liu et al. “Molecular architecture of native HIV-1 gp120 trimers”. In: *Nature* 455.7209 (Sept. 2008), pp. 109–113. DOI: 10.1038/NATURE07159.

[13] Xinzheng Zhang et al. “Structure of Acidic pH Dengue Virus Showing the Fusogenic Glycoprotein Trimers”. In: *Journal of Virology* 89.1 (Jan. 2015), pp. 743–750. DOI: 10.1128/JVI.02411-14. URL: https://journals.asm.org/journal/jvi.

[14] Sai Li et al. “Acidic pH-Induced Conformations and LAMP1 Binding of the Lassa Virus Glycoprotein Spike”. In: (2016). DOI: 10.1371/journal.ppat.1005418. URL: http://www.mrc.ac.uk/.

[15] Audray Harris et al. “Influenza virus pleiomorphy characterized by cryoelectron tomography”. In: *Proceedings of the National Academy of Sciences of the United States of America* 103.50 (2006), pp. 19123–19127. ISSN: 00278424. DOI: 10.1073/pnas.0607614103.

[16] Kay Grünewald et al. “Three-Dimensional Structure of Herpes Simplex Virus from Cryo-Electron Tomography”. In: *Science* 302.5649 (Nov. 2003), pp. 1396–1398. ISSN: 0036-8075. DOI: 10.1126/SCIENCE.1090284. URL: https://science.sciencemag.org/content/302/5649/1396%20https://science.sciencemag.org/content/302/5649/1396.abstract.

[17] Daniel R. Beniac et al. “Architecture of the SARS coronavirus prefusion spike”. In: *Nature Structural and Molecular Biology* 13.8 (Aug. 2006), pp. 751–752. ISSN: 15459993. DOI: 10.1038/nsmb1123.

[18] Montserrat Bá Rcena et al. *Cryo-electron tomography of mouse hepatitis virus: Insights into the structure of the coronavirion*. Tech. rep. 2. 2009, pp. 582–587.

[19] Seiya Ozono et al. “SARS-CoV-2 D614G spike mutation increases entry efficiency with enhanced ACE2-binding affinity”. In: *Nature Communications* 12.1 (2021). ISSN: 20411723. DOI: 10.1038/s41467-021-21118-2. URL: http://dx.doi.org/10.1038/s41467-021-21118-2.

[20] Jun Zhang et al. “Structural impact on SARS-CoV-2 spike protein by D614G substitution”. In: *Science* 372.6541 (2021), pp. 525–530. ISSN: 10959203. DOI: 10.1126/science.abf2303.

[21] Xiao-Hong Nancy Xu, Zhaoyang Wen, and William J Brownlow. “Ultrasensitive Analysis of Binding Affinity of HIV Receptor and Neutralizing Antibody Using Solution-Phase Electrochemiluminescence Assay”. In: *J Electroanal Chem (Lausanne Switz)* 688 (2013), pp. 53–60. DOI: 10.1016/j.jelechem.2012.08.004. URL: www.odu.edu/sci/xu/xu.htm;Tel/fax:.

[22] Yen-Lung Lo et al. “Dengue Virus Infection Is through a Cooperative Interaction between a Mannose Receptor and CLEC5A on Macrophage as a Multivalent Hetero-Complex”. In: (2016). DOI: 10.1371/journal.pone.0166474.

[23] Jason Botten et al. “Identification of Protective Lassa Virus Epitopes That Are Restricted by HLA-A2”. In: *Journal of Virology* 80.17 (Sept. 2006), pp. 8351–8361. DOI: 10.1128/JVI.00896-06.

[24] Huafeng Xu and David E. Shaw. “A Simple Model of Multivalent Adhesion and Its Application to Influenza Infection”. In: *Biophysical Journal* 110.1 (2016), pp. 218–233. ISSN: 15420086. DOI: 10.1016/j.bpj.2015.10.045. URL: http://dx.doi.org/10.1016/j.bpj.2015.10.045.

[25] Na Zhang et al. “Binding of herpes simplex virus glycoprotein D to nectin-1 exploits host cell adhesion”. In: *Nature Communications* 2.1 (2011). ISSN: 20411723. DOI: 10.1038/ncomms1571.

[26] Hoang Linh Nguyen et al. “Does SARS-CoV-2 bind to human ACE2 more strongly than does SARS-CoV?” In: *Journal of Physical Chemistry B* 124.34 (2020), pp. 7336–7347. ISSN: 15205207. DOI: 10.1021/acs.jpcb.0c04511.

[27] Guiqing Peng et al. “Crystal structure of mouse coronavirus receptor-binding domain complexed with its murine receptor”. In: *Proceedings of the National Academy of Sciences of the United States of America* 108.26 (2011), pp. 10696–10701. ISSN: 00278424. DOI: 10.1073/pnas.1104306108.
